# Feeding the night shift: timing, cortisol, and what to do next

**DOI:** 10.1093/sleep/zsaf307

**Published:** 2025-10-02

**Authors:** Minghui Wang, Marie-Pierre St-Onge

**Affiliations:** Mailman School of Public Health, Columbia University Irving Medical Center, NY 10032, USA; Mailman School of Public Health, Columbia University Irving Medical Center, NY 10032, USA; Division of General Medicine and Center of Excellence for Sleep & Circadian Research, Department of Medicine, Columbia University Irving Medical Center, New York, NY 10032, USA

*For night shift workers, no overnight calories may best preserve the cortisol nadir: even a modest snack raises overnight cortisol concentrations and blunts its crucial morning rise over time.*

Cortisol is both a marker of circadian rhythms and metabolic health. Its diurnal rhythm is sensitive to environmental factors changing in night shift, such as light and sleep duration [1]. Night eating (NE) is also common among night shift workers to alleviate hunger, stay alert or relieve gastrointestinal discomfort. Since meal timing entrains peripheral clocks, NE may desynchronize metabolic rhythms from the suprachiasmatic nucleus (SCN)’s rest–fast program, especially because human appetite peaks in the late evening and declines overnight [2, 3]. However, it is unclear how NE itself independently affects cortisol during night shifts. Previous research shows that restricting food intake to the biological day reduces internal circadian misalignment, negative metabolic outcomes, and cardiovascular disease during simulated night work [4, 5]. These observations motivate the exploration of NE’s impact on cortisol to uncover its role in circadian misalignment and downstream health.

In this issue, Grosser et al. test how NE alters the cortisol profile across four simulated night shifts [6]. After an adaptation night, 52 healthy adults were randomized to one of three NE strategies occurring at 00:30 h (meal, snack, or no-meal) with the 4-day menus across the different conditions matched on energy and macronutrient composition. All conditions provided 30% of daily energy at breakfast (07:00 h) and 40% at dinner (19:00 h). The remaining 30% of daily energy was consumed entirely at 00:30 h in the meal condition while in the snack condition 10% of daily energy was consumed at 00:30 h (snack) and 20% at an additional predinner snack (upon waking during the day, 17:30 h) and in the no-meal condition, nothing was consumed at 00:30 and the 30% was reallocated as 10% as a morning predaytime sleep snack (09:30 h) and 20% as an additional predinner snack (same as in the snack condition; upon waking during the day, 17:30 h). This resulted in four eating occasions in the snack and no-meal conditions and three in the meal condition. Salivary cortisol was sampled hourly from 20:00 to 05:30 h, with additional samples at 30, 60, and 120 min after the 00:30 meal or snack (or lack thereof for the no-meal condition). Sleep was measured using wrist-actigraphy.

Across four night shifts, evening cortisol was higher while morning levels were lower on night 4 versus night 1(*p* = .007; *p* = .003), regardless of intervention condition. NE, compared to no nighttime consumption, increased cortisol secretion (meal *p* = .019; snack *p* = .005). Post-intake rises were transient in the meal condition (night 2 only) but persisted across the full duration of the intervention in the snack condition compared to the no-meal condition. However, no differences were noted between snack and meal conditions across the intervention period. The study concluded that night shifts alter the cortisol rhythm while NE increases cortisol concentrations; repeated disruptions may have cumulative effects.

The study had numerous strengths. First, the study employed isocaloric, composition-matched menus, and repeating simulated night shifts with fixed work and sleep times that were consistent across interventions [5]. Second, using hourly saliva assays to measure free cortisol avoided the corticosteroid-binding globulin-driven variability inherent to total serum cortisol, reflecting the biologically active fraction [7]. Third, total sleep time and sleep efficiency did not differ between conditions, avoiding systematic bias from differential sleep duration [8].

However, four features of the study complicate its interpretation. First, although the sleep period was identical in all conditions, sleep was reduced relative to the participants’ usual sleep. While a limitation, this provides ecological validity to the study as most shift workers sleep less on days of night shift compared to day shift [9]. Second, this trial does not truly isolate “meal timing” as the single moderator of the cortisol response. The snack condition had a longer eating window than the meal and no-meal conditions. It is possible that the persistent elevation in postprandial cortisol levels in the snack condition could be due to the longer eating window in that condition. Indeed, the meal and no-meal conditions had the shortest eating window (~12 h), at inverted hours, nighttime for the meal condition and daytime for no-meal condition, whereas the snack condition had approximately 1.5 h longer eating window. Furthermore, both nighttime eating conditions were at the same time, therefore the study more accurately tested the influence of the quantity of calories consumed at a single point in time at night rather than the timing of intake. As such, the study cannot provide information on which to determine an optimal time of food intake for those working at night. Third, eating occasion frequency may confound the results: the snack and no-meal conditions each had four eating occasions, while the meal condition had three. Each eating occasion triggers an acute cortisol surge; thus, higher eating frequency increases the number of postprandial peaks [10]. Although the net effect of eating frequency on total 24-h cortisol exposure depends on single-meal size and total energy intake, under night shift contexts, repeated postprandial states may plausibly amplify adverse responses [8]. Finally, cortisol was sampled only from 20:00 to 05:30 h in the current study, which prevents 24-h inference and limits conclusions about unmeasured periods.

During night work, both feeding window length and meal frequency shape cortisol. Shorter overnight fast and more eating occasions add food exposures and postprandial pulses, whereas sustained fast helps preserve the early morning nadir [11, 12]. Meal timing also acts as a zeitgeber for peripheral clocks. Delaying the main meal toward evening increases evening cortisol [13] and, in simulated night work, confining intake to the biological day maintains internal alignment and prevents the glucose intolerance seen with NE [4, 5].

To truly answer the question of when night shift workers should eat to optimize cortisol timing/exposure and circadian alignment (a stable phase relation between the SCN and peripheral/behavioral rhythms), future studies should hold other timing factors constant and change only one factor at a time: (1) time of NE: compare an earlier vs later main meal of identical composition within the same eating window [14]; in non-shift settings, a later dinner has been shown to raise evening cortisol by 5% [13]; (2) length of the eating window: test a narrow (e.g. 8 h) vs extended (e.g. 12 h) window during wake; time-restricted eating has been linked to changes in cortisol rhythmicity [12, 14]; (3) NE size and frequency: since high-calorie meals cause an immediate cortisol rise, compare frequent small snacks with fewer larger meals under matched total energy and window across conditions [15]; (4) NE schedule consistency or alignment with the biological day: irregular eating patterns may weaken entrainment and daytime-only eating during simulated night work preserves internal alignment and prevents the glucose intolerance seen with NE [4, 16]. Such designs would isolate the impact of timing and frequency of eating on metabolic outcomes.

Importantly, light can also influence the generalizability of this study. Nocturnal light may suppress the amplitude of cortisol rhythm; similar weakening was also observed in another simulated night shift protocol using ~100 lux during the biological night [1]. Yet, dim-light forced desynchrony showed little suppression during circadian misalignment regardless of feeding time [4]. Light can also acutely increase cortisol [17, 18]. Future research should measure the spectral composition and explore light×NE interactions. Finally, measurement only during wake (~10 h/night) limited full diurnal characterization. Without a 24-h profile, amplitude and acrophase cannot be estimated accurately.

Implications of this work for future research endeavors and clinical practice are important. The findings set the stage for additional research with expanded outcomes based on chrononutrition strategies to improve the health of night shift workers. This is relevant for public health research since high night time and a flatter daily rhythm of cortisol are linked to more insulin resistance and higher diabetes risk [19]. Only with added information, including more outcomes, in research performed in ecological settings, can we raise awareness of the health risks of night shift work. Studying potential prevention strategies such as timing, frequency, and quality of meals should be a main area of focus to strengthen protections for workers’ health and rights [20].
